# A Clinical Treatise on Diseases of the Breast

**Published:** 1899-09

**Authors:** 


**A Clinical Treatise on Diseases of the Breast.** By A. Marmaduke Sheild, M.B. Pp. xvi., 510. London: Macmillan and Co., Limited. 1898.

It would be a mistake to think all pathology had been omitted from Mr. Sheild's book because he calls it a "clinical treatise," but the work is eminently one for the guidance of the surgeon in the many difficulties he has to encounter both in the diagnosis and the treatment of diseases of the breast. Mr. Sheild has written an admirable and a very readable book—the style is good, and the information clearly given. Coloured plates abound, and we have hardly ever seen morbid conditions so clearly represented. We must, however, make an exception of Plate XIV., which we think does not represent the scirrhus-growth as nearly white enough.

The results of operations for cancer of the breast are given with a clearness and fulness which make this part of the work of much value. This we might expect, for it is not long since Mr. Sheild brought the matter before the Royal Medical and Chirurgical Society, and elicited the opinions and experiences of both the senior and junior surgeons in London, some of whom adopted very different lines of practice. We cannot help saying that we consider there is one fault in the book, which, however, is not confined to Mr. Sheild's writings or sayings. It is the idea implied now and again in this work that a London surgeon has special opportunities for arriving at a right conclusion on some disputed facts. This all depends on the amount of clinical or pathological material available by the individual surgeon. Many surgeons attached to large provincial hospitals have much greater experience of diseases than surgeons on the staff of some of the London hospitals.

All that is best in recent periodical literature on the subject on which Mr. Sheild writes is referred to by him. Duct cancer, a subject about which so much has been written of late, and of which various descriptions are still given, is clearly described, and its distinction (which is so great) from ordinary cancer is well given. Beatson's method of treating cancer by oöphorectomy receives some notice, and also the treatment of malignant disease of the breast by Coley's fluid. Reference is of course made to Mr. Gould's very interesting case, in which an undoubted recurrent cancer of the breast and glands entirely disappeared. Mr. Sheild is sceptical as to the spontaneous disappearance of fibro-adenomata of the breast. He thinks such swellings have probably been sclerosing mastitis or cysts with indurated breast-tissue around. He says fibro-adenomata roll about in the breast. This is a very common statement; but we should like to know how the movement takes place. There is no space for them to roll about in. The finger receives the sensation as if they were rolling about in the breast, but that is because the part of the breast in which, or on which, they lie can be so readily moved on the other portions of the organ. A full and instructive account is given of Paget's disease of the nipple, or "dermatitis maligna" as Mr. Sheild calls it, in which the relation of the disease to psorosperms is discussed.

We can strongly recommend the book, not only to the busy practitioner who needs to refer to some modern work for guidance in dealing with diseases of the breast, but also to the operating surgeon, for the problems which he has to face are clearly and ably discussed.

